# Tailoring Interfacial Exchange Anisotropy in Hard–Soft Core-Shell Ferrite Nanoparticles for Magnetic Hyperthermia Applications

**DOI:** 10.3390/nano12020262

**Published:** 2022-01-14

**Authors:** Venkatesha Narayanaswamy, Imaddin A. Al-Omari, Aleksandr S. Kamzin, Bashar Issa, Ihab M. Obaidat

**Affiliations:** 1 Department of Medical Diagnostic Imaging, College of Health Sciences, University of Sharjah, Sharjah 27272, United Arab Emirates; venkateshnrn@gmail.com; 2 Department of Physics, Sultan Qaboos University, Muscat 123, Oman; ialomari@squ.edu.om; 3 Laboratory of Ferroelectricity and Magnetism Physics, Ioffe Physical Technical Institute, 194021 St. Petersburg, Russia; askam@mail.ioffe.ru; 4 Department of Physics, United Arab Emirates University, Al-Ain 15551, United Arab Emirates

**Keywords:** exchange bias, exchange anisotropy, SAR, magnetization, hyperthermia

## Abstract

Magnetically hard–soft core-shell ferrite nanoparticles are synthesized using an organometallic decomposition method through seed-mediated growth. Two sets of core-shell nanoparticles (S1 and S2) with different shell (Fe_3_O_4_) thicknesses and similar core (CoFe_2_O_4_) sizes are obtained by varying the initial quantities of seed nanoparticles of size 6.0 ± 1.0 nm. The nanoparticles synthesized have average sizes of 9.5 ± 1.1 (S1) and 12.2 ± 1.7 (S2) nm with corresponding shell thicknesses of 3.5 and 6.1 nm. Magnetic properties are investigated under field-cooled and zero-field-cooled conditions at several temperatures and field cooling values. Magnetic heating efficiency for magnetic hyperthermia applications is investigated by measuring the specific absorption rate (SAR) in alternating magnetic fields at several field strengths and frequencies. The exchange bias is found to have a nonmonotonic and oscillatory relationship with temperature at all fields. SAR values of both core-shell samples are found to be considerably larger than that of the single-phase bare core particles. The effective anisotropy and SAR values are found to be larger in S2 than those in S1. However, the saturation magnetization displays the opposite behavior. These results are attributed to the occurrence of spin-glass regions at the core-shell interface of different amounts in the two samples. The novel outcome is that the interfacial exchange anisotropy of core-shell nanoparticles can be tailored to produce large effective magnetic anisotropy and thus large SAR values.

## 1. Introduction

Tuning the magnetic properties of nanoparticles for enhanced biomedical applications such as magnetic resonance imaging (MRI) contrast agents, magnetic particle imaging, drug delivery, biosensing, magnetic marker-based bioassay, and magnetic hyperthermia has been studied intensively [1,2,3]. Parameters of the nanoparticles such as the size, shape, composition, and interface in core-shell nanoparticles were employed to enhance the efficiency in both diagnosis and treatment of cancer [4,5]. Manipulating the magnetic properties through the interface coupling of different magnetic phases provides a new variable for rational material design and controlling the properties for fundamental science and technological applications [6,7]. The usual strategies used to tune the magnetic properties of nanoparticles are varying the size, shape, and composition, whereas having bi-magnetic phases in the nanoparticles provides the combined effect of multiphase properties [8,9]. The ability to combine novel properties of different materials and to fabricate nanostructures with improved efficiency increased interest in core-shell architecture [10]. Recent reports suggested that bi-magnetic core-shell nanocrystals can improve the energy production of the magnetic nanoparticles beyond the superparamagnetic limit for applications, particularly in magnetic storage [11]. The magnetic properties of core-shell structures are usually determined by several parameters such as the size, order (soft–hard or hard–soft), and geometric shape of the core and shell [12,13,14]. Furthermore, the magnetic properties are affected by differences in magnetic parameters between the core and shell materials, as well as the presence of dipolar and exchange-coupled interactions that affect spin reversal processes [15]. The distribution of particle sizes and morphology of the core-shell structures are also important factors determining their magnetic properties [16,17]. In biomedicine, magnetic nanoparticles can be used in magnetic resonance imaging (MRI) as contrast agents and in magnetic hyperthermia as heat sources [18,19]. The conversion of electromagnetic energy into heat by nanoparticles has the potential to be a powerful non-invasive technique for biomedical applications such as drug release, cancer treatment, and remote control of single cell functions, but poor conversion efficiencies have so far hampered practical applications [20,21]. The magnetic nanoparticles convert electromagnetic energy to heat when excited with an AC field, and the heat produced is used to kill cancer cells and harmful bacteria. The nanoparticles produce heat via several processes such as eddy currents, frictional heating, magnetic NP relaxation (Neel and Brownian), and hysteresis losses [22]. Using the linear response theory (LRT), the conversion efficiency of magnetic energy into heat energy of the superparamagnetic nanoparticles is expressed in terms of the specific adsorption rate given by Equation (1) [23].

(1) $$\mathrm{SAR}=\mu_{0}{\mathrm{fH}}_{0}^{2}{\pi \chi }_{0\;}\frac{2{\pi f\tau }_{\mathrm{eff}}}{1+{\left(2{\pi f\tau }_{\mathrm{eff}}\right)}^{2}}\frac{1}{\rho }$$

SAR values of the given nanoparticles depend on several external factors such as magnetic field amplitude (H_0_), AC frequency (f), and intrinsic properties such as DC susceptibility ($\chi_{0\;}$), effective relaxation time ($\tau_{\mathrm{eff}}$), saturation magnetization (M_S_), and the density of magnetic nanoparticles (ρ). Tuning these properties of the nanoparticles will enhance the efficiency of the nanoparticles for magnetic hyperthermia. Core-shell nanoparticles are studied for potential applications in magnetic hyperthermia with higher efficiency to minimize the dose level to kill cancer cells and prevent collateral damage of healthy cells. Several research groups reported SAR of various compositions and geometry-dependent nanoparticles to explore the interface effect for magnetic heating [24,25,26]. Magnetic coupling across the interface of the different magnetic phases provides the NPs with exchange bias (EB) properties that helps in the efficient conversion of electromagnetic energy into heat for magnetic hyperthermia [27]. However, the effect of the core-shell geometry on the magnetic anisotropy and magnetization dynamics such as Neel and Brownian relaxations is not clearly understood. The work presented here involves the synthesis of core-shell nanoparticles with the same core size and two different shell thicknesses. Core-shell nanoparticles were synthesized using the seed-mediated method by employing two different initial concentrations of seed nanoparticles in the organometallic decomposition method. A different shell thickness was obtained because of the difference in the nucleation and growth rates that were mediated by the concentration and diffusion rates of the precursors. The magnetic properties such as the exchange bias, saturation magnetization, coercivity, and remnant magnetization were determined from the MH data. These parameters were used to understand the effect of the magnetic anisotropy on the heating ability of the nanoparticles. The SAR values of the core-shell nanoparticles were determined in agar ferrogel phantom. The EB and SAR results were discussed and attributed to the occurrence of spin-glass-like regions across the core-shell interface. The nanoparticles showed high SAR values and strong dependency on the shell thickness. Hence, designing these sets of nanoparticles will help to enhance the magnetic hyperthermia efficiency for cancer treatment.

## 2. Materials and Methods

### 2.1. Synthesis of CoFe_2_O_4_ Nanoparticles

Ferrite nanoparticles used in this study were synthesized using the well-reported organometallic decomposition method [28]. CoFe_2_O_4_ nanoparticles were synthesized by adding Co(acac)_2_ (257.0 mg), Fe(acac)_3_ (706.4 mg), 1,2-hexadecanediol (40 mg), oleic acid (2 mL), and oleyl amine (2 mL), and diphenyl ether (20 mL) to a round-bottom flask. The round-bottom flask was fitted with a reflux condenser and magnetic stirrer, and the solution mixture was heated to 150 °C under an argon atmosphere for 30 min. The temperature of the reaction mixture finally increased to 250 °C and was heated for 60 min, after which the synthesized nanoparticles were cooled to room temperature. At room temperature, 50 mL ethanol was added to the reaction mixture to separate the nanoparticles. The nanoparticles were further washed with a mixture of hexane and ethanol using a centrifuge at 9000 rpm.

### 2.2. Synthesis of Core-Shell Nanoparticles

Two sets of CoFe_2_O_4_-Fe_3_O_4_ core-shell nanoparticles were synthesized using 20 (S1) and 40 (S2) mg of CoFe_2_O_4_ nanoparticles synthesized in the first step as seeds. The measured quantities (20 and 40 mg) of seed nanoparticles were dispersed in 30 mL of hexane by sonication for 10 min. To this particle dispersion, 20 mL of benzyl ether, Fe(acac)_3_ (1059.6 mg), oleic acid (2 mL), oleyl amine (2 mL), and 1,2-hexadecanediol (40 mg) were added. The reaction mixture was heated to 100 °C for 60 min to evaporate the hexane. The temperature of the reaction mixture was then increased to 150 °C , which was maintained for 30 min. The temperature of the reaction mixture was finally increased to 290 °C and heated for 60 min under argon flow. The reaction mixture containing synthesized nanoparticles was cooled to room temperature. Then, 50 mL ethanol was added to the reaction mixture to sediment the nanoparticles that were subsequently washed using an ethanol and hexane mixture.

### 2.3. Characterization of the Nanoparticles

Structural phases and the average crystalline sizes of the nanoparticles were determined from the X-ray diffraction pattern using a Shimadzu-6100 powder XRD diffractometer fitted with Cu-Kα radiation (wavelength 1.542 Å). The XRD patterns were recorded in the range of 20 to 70° 2θ. A Titan Themis 300 kV from an FEI transmission electron microscope (TEM) was used to obtain the bright-field images and selected area electron diffraction patterns. The nanoparticle size distributions were obtained using ImageJ software. The dc magnetic measurements were carried out using a VSM in the Physical Properties Measurement System (PPMS) from Quantum Design.

### 2.4. Magneto Thermal Measurements

The heating profiles of nanoparticles were obtained using a nanoScale Biomagnets hyperthermia instrument. In calorimetric measurements, an AMF was applied to the ferrogel of MNPs, and the increase in the temperature was measured with respect to time. The SAR values were obtained from the initial slope of the (temperature–time curve)-measured data using Equation (2) [29]:

(2) $$\mathrm{SAR}\;\left(W/g\right)=\frac{C}{m_{\mathrm{MNP}}}\frac{\mathrm{dT}}{\mathrm{dt}}$$

where C (in J/K) is the heat capacity of the ferrogel (which includes the MNPs and the agar gel), $m_{\mathrm{MNP}}$ is the mass (in g) of the MNPs in the ferrogel, and $\frac{\mathrm{dT}}{\mathrm{dt}}$ is the initial slope of the temperature–time curve. The heat capacity of the sample is the sum of the specific heat capacities multiplied by the mass of the components (MNPs, water, and agar) of the ferrogel.

## 3. Results and Discussion

### 3.1. Structural and Magnetic Characterization of CoFe_2_O_4_ Nanoparticle Seeds

**Figure 1:**
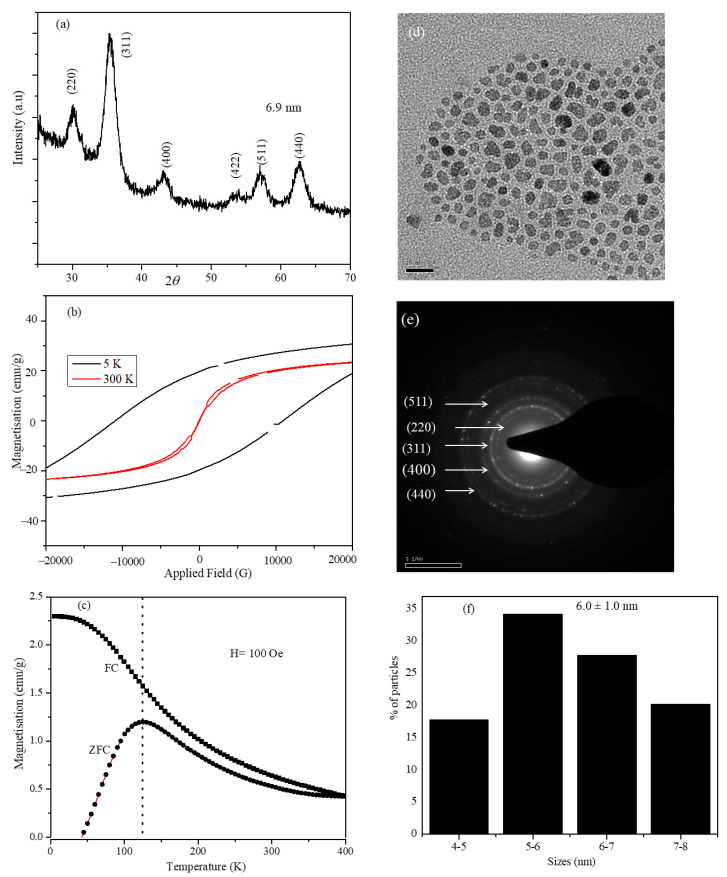
(**a**) XRD pattern, (**b**) Field-dependent magnetization hysteresis loops at 5 and 300 K, (**c**) ZFC and FC temperature-dependent magnetization plots with field 100 Oe, and (**d**–**f**) Bright-field TEM image, SAED pattern, and size distribution of CoFe_2_O_4_ nanoparticles.

The XRD profile of the CoFe_2_O_4_ nanoparticles is shown in Figure 1a; the average particle size obtained from the highest intensity peak (311) was 6.9 nm. The average particle sizes of CoFe_2_O_4_ seed nanoparticles were determined using the Scherrer equation. The FWHM of the (311) peak was determined by a Gaussian fitting function using Origin software. All diffraction peaks were indexed to a cubic spinel phase of CoFe_2_O_4_ with space group Fd3m (JCPDS Card No 22-1086). The lattice constant was determined by multiple peak fitting of the XRD pattern (8.376 Å). TEM bright-field images, SAED pattern, and the size distribution plot are shown in Figure 1d–f. XRD and SAED patterns indicate that the nanoparticles synthesized contained only the ferrite phase, and the lattice parameters are in agreement with the reported values of CoFe_2_O_4_ nanoparticles [30,31]. CoFe_2_O_4_ nanoparticles have an irregular shape and considerable size distributions with an average size of 6.0 ± 1.0 nm. Magnetization vs. applied field was measured at room temperature and at 5 K, and the plots are shown in Figure 1b. Temperature-dependent magnetization under ZFC and FC conditions was measured at 100 Oe, and the plots are shown in Figure 1c. The MH plot at room temperature shows both zero coercivity and zero remnant magnetization, which indicates the superparamagnetic nature of the seed nanoparticles. The ZFC plot also confirms this, where it shows a blocking temperature around 125 K, and nanoparticles are superparamagnetic above this temperature.

### 3.2. Structural and Magnetic Characterization of Core-Shell Nanoparticles

**Figure 2:**
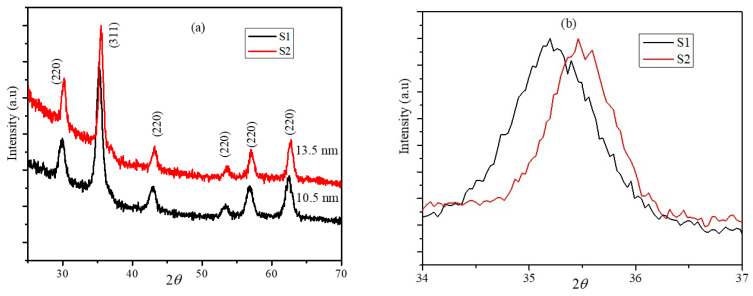
(**a**) XRD of the core-shell nanoparticles S1 and S2. (**b**) Shift in the highest intensity peak (311) of the S1 and S2 nanoparticles.

The XRD diffraction patterns of core-shell nanoparticles S1 and S2 are shown in Figure 2a. The core-shell nanoparticles retained the ferrite phase and did not have any additional peaks corresponding to the other iron oxides. The S1 nanoparticle peaks shifted to the lower diffraction angle with respect to the diffraction pattern of the S2 nanoparticles. The core-shell nanoparticles exhibited a cubic spinel structure (Fd3m) with the shift in the 2theta values because of the co-existence of the Fe_3_O_4_ and CoFe_2_O_4_ phases. The average crystalline sizes of the core-shell nanoparticles determined from the FWHM of the (311) peak based on the Scherrer equation were 10.5 and 13.5 nm for particles S1 and S2, respectively [32]. The lattice parameters of the seed and core-shell nanoparticles were determined using the UnitCell program, which uses a non-linear least squares method and regression diagnostics [33]. The positions of the (220), (311), (222), (400), (511), and (440) indexed peaks were determined by a Gaussian fitting function using Origin software; these values were used to calculate the lattice constant using UnitCell. The lattice constants of the core-shell nanoparticles were obtained using the multiple peak fitting method and were 8.42 and 8.390 Å for S1 and S2, respectively. The lattice constants of the seed and core-shell nanoparticles are comparable with the reported values of CoFe_2_O_4_ and Fe_3_O_4_ individual nanoparticles. The shift of the highest intensity peak of S1 at around 35° is shown in Figure 2b; this shift is due to the higher phase fraction of CoFe_2_O_4_ compared to the Fe_3_O_4_ phase. The lattice parameters of the nanoparticle systems presented here are compared with the some of the reported values in Table 1.

**Figure 3:**
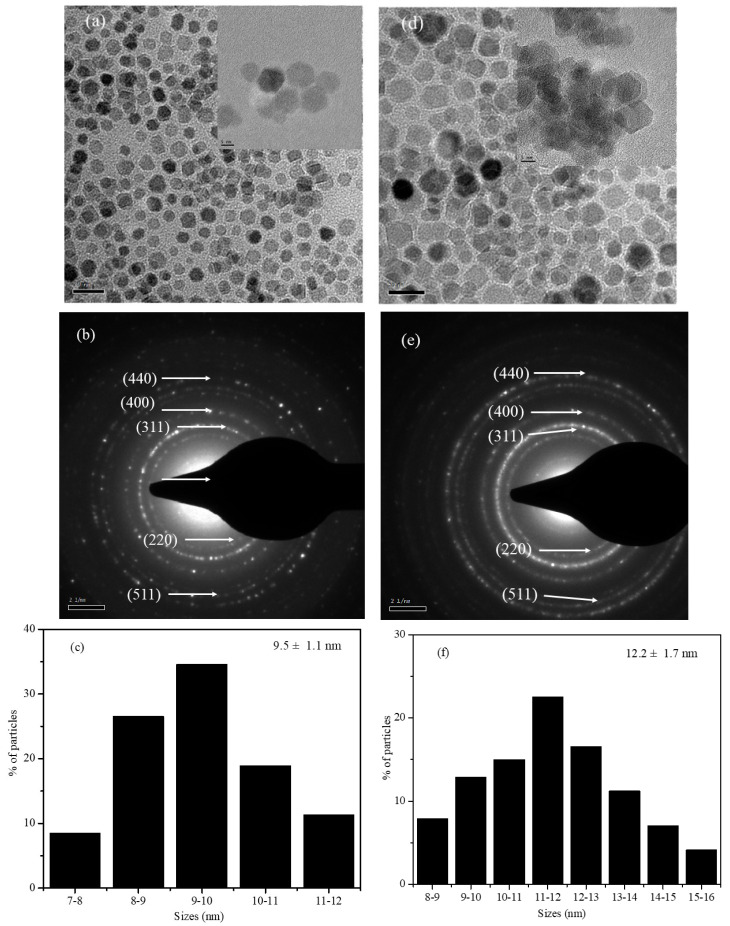
TEM bright-field images, SAED patterns, and size-distribution histograms of the S1 (**a**–**c**) and S2 (**d**–**f**) core-shell nanoparticles.

Bright-field TEM images (HRTEM images are shown as an inset), SAED patterns, and size-distribution histograms of the S1 and S2 nanoparticles are shown Figure 3. TEM images of the S1 nanoparticles showed that the particles were mainly spherical in shape. The shape of the core-shell nanoparticles of S2 comprised spherical and edge-shaped particles, which might be due to the shape of the initial seed nanoparticles used that were irregular and upon the growth of the shell, nanoparticles prefer to have a spherical and cubical geometry. The diffraction patterns of both core-shell nanoparticles agree with the reported patterns of ferrite nanoparticles, which indicates the absence of any other oxide phases [28,36]. From the TEM images of Figure 3, it is observed that the S1 and S2 nanoparticles had significant size distributions that are displayed in the size-distribution histograms. The size distributions shown in Figure 3c,f were obtained by measuring the sizes of more than 200 individual particles from different TEM images. While measuring the size of the individual particles, a good amount of care was taken to avoid overlapped particles and only particles with a well-separated outer boundary were considered. The average size of the nanoparticles was calculated from the histograms as 9.5 ± 1.1 and 12.2 ± 1.7 nm for S1 and S2, respectively. The size distribution histograms with error bars obtained from the TEM images are in good agreement with the average crystalline sizes calculated from XRD. The shell thicknesses of the core-shell nanoparticles were obtained by subtracting the average size of the seed nanoparticle, and the corresponding shell thicknesses of the S1 and S2 nanoparticles were 3.5 and 6.1 nm, respectively.

The elements in the ferrite core and shell nanoparticle systems presented here have very similar atomic numbers, and thus there are no visible differences in the contrast of the TEM and HRTEM bright-field images, and we could not obtain any contrast in the images to identify the core and shell boundaries. However, from the TEM (and HRTEM) images of the seed and core-shell nanoparticles, the sizes of the core-shell nanoparticles were larger than those of the seed nanoparticles. We can also observe that the particles were well separated from each other. The size distributions obtained from the images are an acceptable estimation of the average sizes. In fact, the average sizes obtained from the FWHM of the (311) peaks are also in agreement with the sizes obtained from TEM. The main problem is to confirm the core-shell structure of our samples. To address this issue, we have compared the results with several reports of ferrite core-shell nanoparticle systems using an organometallic decomposition method for the synthesis of core-shell nanoparticles [6,25,26,28,36]. These reports suggest that in the seed-mediated method, the nanoparticles surface (CoFe_2_O_4_) will act as a nucleating site for the nucleation and subsequent growth of the shell phase (Fe_3_O_4_), which is thermodynamically favorable rather than phase separation. This gives support to our claims of core-shell structure since we used a similar synthesis method.

**Figure 4:**
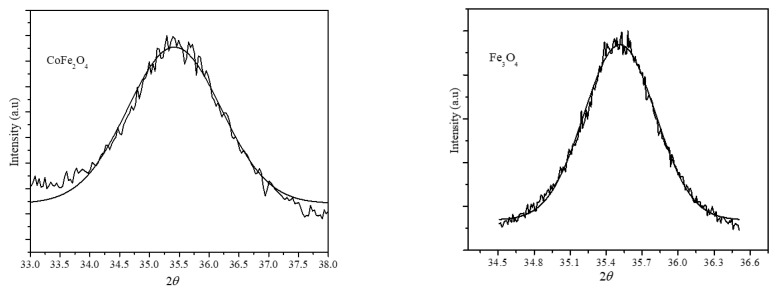

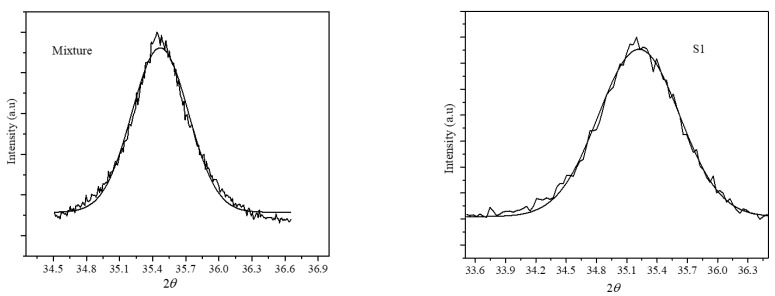
The (311) peak position obtained by Gaussian fitting of the XRD diffraction peak.

We used the shift in the XRD peak position to confirm the formation of the core-shell structure [37]. We obtained the (311) peak position from the diffraction patterns of the individual Fe_3_O_4_, CoFe_2_O_4_ nanoparticles, and the mechanical mixture of Fe_3_O_4_ and CoFe_2_O_4_ nanoparticles (with 1:1 mass ratio). We compared the peak positions with that obtained from the (assumed) core-shell nanoparticles of sample S1. The (311) peak position was obtained in the angle range of 33–38° with a step size of 0.01 and exposure time of 100 s/point. Peak positions were obtained by Gaussian fitting; diffraction data along with the fitted curves are shown in Figure 4, and values are listed in Table 2. The XRD peak positions of the individual CoFe_2_O_4_ and Fe_3_O_4_ nanoparticles were 35.45° and 35.5°, respectively, whereas for the mechanical mixture, the peak position was 35.48°, which indicates the overlapping of the (311) peaks of the individual nanoparticles that were reflected by the peak position and FWHM values. Interestingly, the (311) peak position of the core-shell nanoparticles (S1) was lower than that of the CoFe_2_O_4_ nanoparticles, which is due to the epitaxial growth of the shell phase and subsequent strain at the interface caused by the core-shell geometry, which caused a significant shift in the peak position.

### 3.3. Magnetic Measurements of the S1 and S2 Core-Shell Nanoparticles

**Figure 5:**
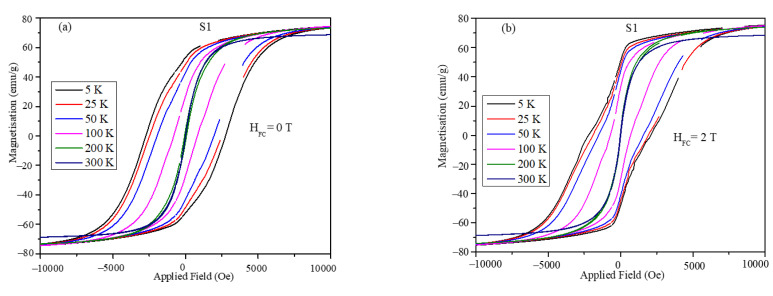

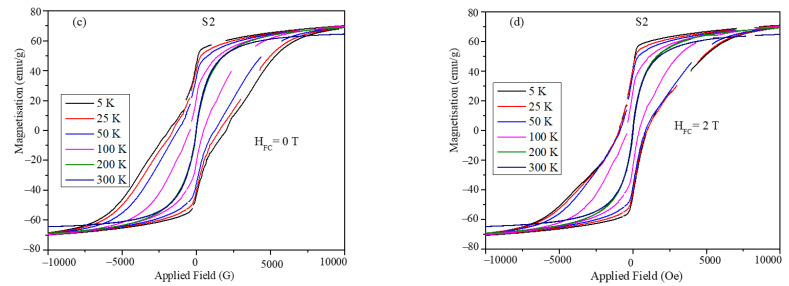
Magnetic hysteresis loops obtained at room temperature in the field range of −10,000.0 Oe to +10,000.0 Oe obtained under the cooling of the 0 and 2 T magnetic field for (**a**,**b**) S1 and (**c**,**d**) S2.

Magnetic hysteresis (MH) loops obtained for the core-shell ferrite nanoparticles are shown in Figure 5. The magnetic hysteresis loops were obtained at temperatures 5, 25, 50, 100, 200, and 300 K at zero-field (ZFC) cooling and at field-cooling (FC) values of 1, 2, and 3 T. The hysteresis loops showed significant openings at low temperatures (below 200 K), whereas at room temperature, the particles were superparamagnetic with negligible remnant magnetization and coercivity. The MH loops in Figure 5 show kinks, which can be attributed to the presence of hard and soft magnetic phase interfaces. The kinks in the case of the S2 nanoparticles were more pronounced compared to those of the S1 particles, which can be attributed to the difference in the phase fractions of the hard (CoFe_2_O_4_) and soft (Fe_3_O_4_) magnetic phases in the two samples.

**Figure 6:**
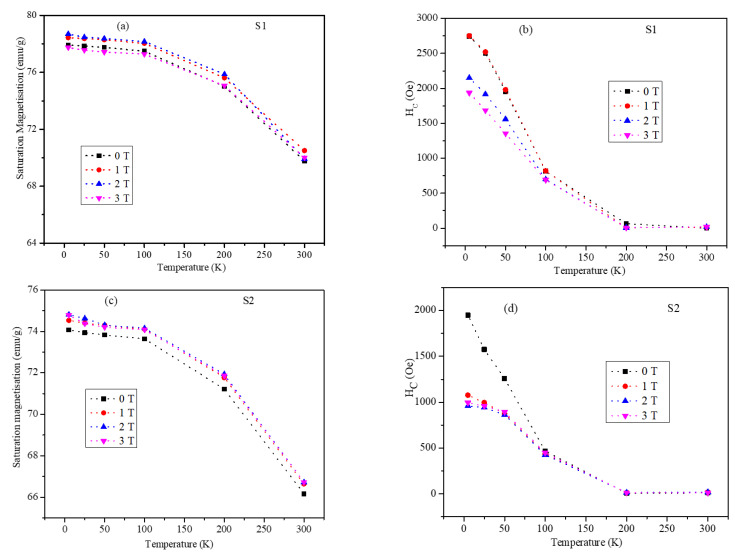
Saturation magnetization as a function of temperature at 0, 1, 2, and 3 T field cooling for (**a**) S1 and (**c**) S2. The coercivity as a function of temperature at 0, 1, 2, and 3 T field-cooling values for (**b**) S1 and (**d**) S2 nanoparticles.

The saturation magnetization (M_S_) of the core-shell nanoparticles obtained at 2 T from the MH plots is shown in Figure 6a,c for samples S1 and S2 in the ZFC state and in the FC state at 1, 2, and 3 T field cooling values. Robles et al. reported the effect of shell thickness on the saturation magnetization in a Fe_3_O_4_-CoFe_2_O_4_ core-shell system. They varied the shell thickness in a controlled way between 1 and 4 nm, and the highest saturation magnetization was obtained for the core-shell geometry with a shell thickness of 2.1 nm [36]. This indicates that in core-shell systems of nanoparticles, the magnetic properties depend on the individual thickness of the magnetic phases rather than on the particle size of the core-shell nanoparticles. In our study, the saturation magnetization values showed a similar trend at all field cooling values. The values of M_S_ were high and nearly constant at low temperature and decreased with the increase in temperature. The observed high and nearly constant values of the saturation magnetization at low temperatures were attributed to the surface spins that were misaligned at room temperature and froze in a disordered structure at lower temperature [38]. S1 nanoparticles had an Ms value of 77.96 emu/g at 5 K and 69.97 emu/g at room temperature, whereas the M_S_ values for the S2 particles were 70 and 66.16 emu/g at 5 and 300 K, respectively. The Ms values of both S1 and S2 particles showed a slight field cooling dependency (except at 4 T in S1 where the field cooling had no effect on M_S_). Considering the commonly observed dominant effect of particle size on the saturation magnetization, S2 particles were expected to have higher M_S_ values. However, we observed considerably lower M_S_ values for S2, which can be attributed to interface effects, as we will discuss later. The saturation magnetization for S1 was greater than that for S2 at all field-cooling values and at all temperatures. For both samples and at all field-cooling values, M_S_ remained nearly constant at temperatures below 100 K and slightly decreased up to 200 K (the spin freezing temperature). M_S_ decreased rapidly at temperatures above 200 K in both samples. However, the rate of decrease of M_S_ in S2 was slightly larger than that in S1.

The coercivity values of the S1 and S2 nanoparticles calculated from the MH plots are shown in Figure 6b,d. The behavior of coercivity with temperature was nearly the same in both samples, but the H_C_ values in the S2 particles were lower than the values in the S1 particles. The coercivity values showed a strong temperature dependency up to 200 K. The coercivity values decreased sharply with increasing temperature up to 200 K and remained flat with negligible magnitudes above 200 K. As can be seen, the H_C_ value varied from 2735 Oe at 5 K to a negligible value above 200 K in S1. The coercivity displayed significant field-cooling dependency at temperatures below 100 K (except at 1 T for S1). For example, at 5 K, the coercivity values for S2 were 1954 and 935 Oe in the ZFC and FC (at 3 T) states, respectively. The highest coercivity values in both samples were obtained at 5 K in the ZFC state. The trends of the saturation magnetization and coercivity of S1 and S2 are interesting. Moon et al. reported a systematic study of the effect of shell thickness on the coercivity value. They reported high coercivity for the ultrathin shell with few atomic layers interface due to the enhanced spin canting [26]. The interface system presented in our study also showed thickness-dependent coercivity.

**Figure 7:**
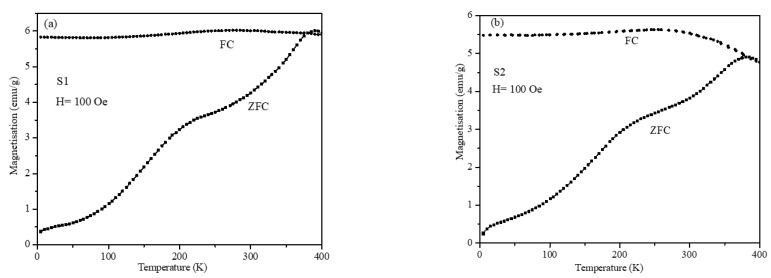
The M-T plots under ZFC and FC conditions with 100 Oe field for (**a**) S1 and (**b**) S2.

The M-T plots under ZFC and FC conditions with the 100 Oe field were obtained for nanoparticles S1 and S2 and are shown in Figure 7. The ZFC plots of both particles showed a smooth peak around 200 K and another well-defined peak at nearly 380 K. Our core-shell nanoparticles with SG-like phase at the interface was composed of two different magnetic regimes. We believe the smooth peak at the lower temperature (around 200 K) was due to the SG-like regime and the well-defined peak at the higher temperature (around 380 K) was due to ferrimagnetic regime of the nanoparticles. The FC and ZFC plots of the core-shell nanoparticles were broad compared with the single-phase seed nanoparticles (Figure 1), which can be attributed to the size distributions and bi-magnetic interface of the two phases. The flattening of the FC magnetization at low temperatures is suggested to be a signature for the occurrence of the spin glass state in magnetic nanoparticles [39]. This feature is clearly observed in FC branches of both samples, S1 and S2, at temperatures below 200 K. The separation of the ZFC and the FC branches can be attributed to significant inter-particle interactions.

**Figure 8:**
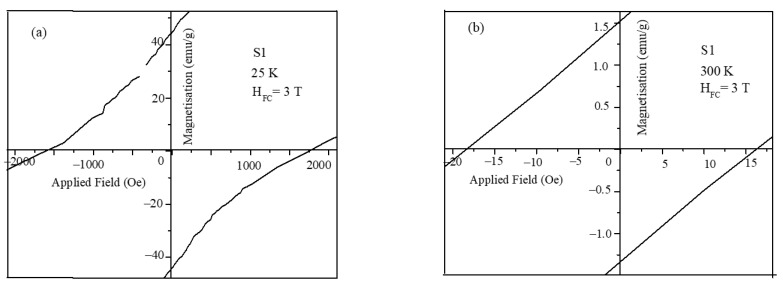
Zoomed portion of MH plots used for exchange bias calculations (**a**) S1 at 25 K under 3Tand (**b**) S1 at 300 K under 3T.

The exchange bias values of the core-shell nanoparticles were obtained from the hysteresis loops of Figure 5. To calculate the exchange bias values and coercivity of the nanoparticles, the shift in the MH loops at the origin under different field cooling was used. The zoomed portion of the MH plots are shown in Figure 8 from which the shift in the magnetization and field values was obtained.

The horizontal shift in the hysteresis loops was defined as the exchange bias field, H_EB_. The exchange bias field H_EB_ was calculated using Equation (3) [40]:

(3) $${\;H}_{\mathrm{EB}}=\frac{H_{C1}+H_{C2}}{2}\;$$

Here, the coercive field at the descending branch of the hysteresis loop is $H_{C1}$, and that on the ascending branch is $H_{C2}$.

**Figure 9:**
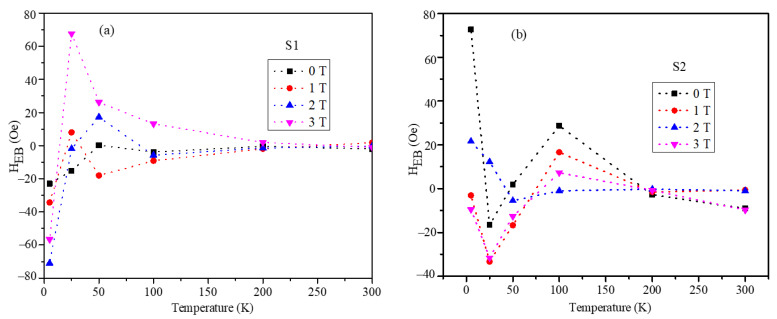
The temperature dependence of H_EB_ in the temperature range of 2–300 K for (**a**) S1 (**b**) S2.

The temperature-dependent exchange bias field values are shown in Figure 9a,b for the S1 and S2 nanoparticles. The exchange bias field values showed non-monotonic trends with temperature in both samples. Above 200 K, the exchange bias field values were negligible for the S1 and S2 nanoparticles. The exchange bias values showed slight dependency on the cooling field though the trends were non-monotonic.

It is interesting that numerous research studies revealed the occurrence of (unconventional) EB. For example, unconventional EB with T_N_ > T_C_ has been observed in some systems such as core-shell nanoparticles and thin-film heterostructures [41,42,43,44]. In addition, EB was reported to occur in systems cooled from temperatures below T_N_ [45]. In [46], the EB effect was found to occur under zero-field cooling. In [47,48,49,50,51,52,53,54], the EB effect was reported in AFM/SG systems. In [49,50,55], a spin glass (SG), such as disorder, was suggested to occur at the FM-AFM interface. In [45], spin clusters such as SG states were suggested to present at the interface of layered polycrystalline thin films. According to the unconventional EB reports with SG materials or a spin SG-like phase, the EB can occur when cooling through the transition temperature of the FIM phase or when cooling through the SG freezing temperature (TSG) [47,48]. We believe that the occurrence of EB in our core-shell nanoparticles is not due to the direct exchange coupling between the FIM core and FIM shell materials but rather is due to the exchange coupling between the FIM-SG phases. Hence, the FIM-FIM exchange coupling is mediated by the FIM-SG interactions. Hence, cooling down from 300 K is sufficient for the EB effect to take place in our samples.

The broken exchange bonds, strain, and the low symmetry at the surface and interface of core-shell NPs result in interfacial uncompensated spins, which could freeze at low temperatures, leading to interface spin-glass (SG) regions with random spin orientations [56]. SG regions that are randomly spread at the layered FM–AFM and core-shell nanoparticle interfaces were suggested to affect the exchange bias properties [57]. The direct exchange interaction between the core and shell spins was prevented by the existence of interfacial SG regions. Each one of the SG regions had a net magnetic moment (spin). The spins of all SG regions were randomly oriented. In addition to the magnetic interaction among the SG regions, they were also coupled to the core and shell of the NP with variable coupling strengths. Hence, based on the spin-glass features of the FC temperature dependence of magnetization and based on our earlier findings on core-shell NPs [57], we suggest that the nonmonotonic and oscillatory behavior of the exchange bias observed in our samples can be attributed to the occurrence of spin-glass regions at the core-shell interface. When the net exchange coupling of all SG regions with the core is antiferromagnetic, positive exchange bias occurs, whereas when the net exchange coupling of all of the SG regions with the core is ferromagnetic, negative exchange bias occurs [58,59]. The whole shell material is involved in the interfacial exchange coupling as indicated from the variations of exchange bias with shell thickness. The temperature and field affect the exchange coupling in an unpredicted manner based on the variable magnetic coupling among the SG regions and between the SG regions and the core and shell spins at the interface. The larger the magnitude of the exchange bias field, the stronger the indirect interfacial exchange bias. As expected, the interfacial exchange coupling strength diminishes with increasing temperature, as can be seen in Figure 8. The oscillatory behavior of the exchange bias field was more prominent in S2, which indicates that the SG regions were larger than those in S1.

The magnetic anisotropy values are significant in understanding the observed coercivity, exchange bias, and conversion of magnetic energy into heat for magnetic hyperthermia. The law of approach to saturation (LAS), which defines the dependency of the magnetization (M) on the applied magnetic field (H) at high field strengths, was employed to calculate the effective anisotropy constant (K_eff_) of the particles. According to the LAS, the magnetization near the saturation is represented using Equation (4). To calculate K_eff_ values, the experimental curves of M as a function of 1/H^2^ were fitted at high magnetic field strengths to obtain M_S_ and fitting parameter b

(4) $$M=M_{s}\left[1-\frac{b}{H^{2}}\right]$$

where parameter b is associated with K_eff_ given by Equation (5)

(5) $${\;K}_{\mathrm{eff}}=\mu_{0}M_{s}\sqrt{\frac{15b}{4}}$$

**Figure 10:**
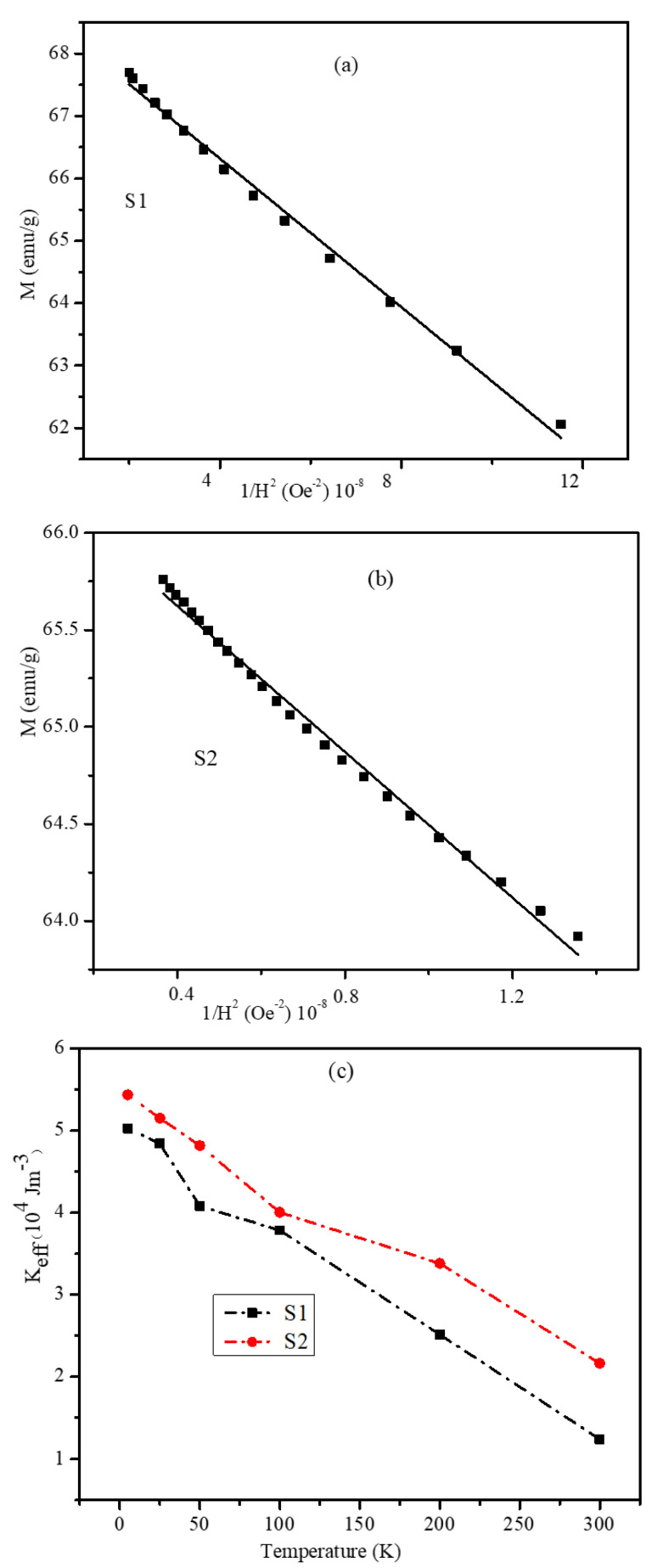
M vs. 1/H^2^ plots obtained at high field strengths for (**a**) S1, (**b**) S2 nanoparticles from the room temperature MH plots. (Experimental data are marked by symbols, and the solid lines represent a linear fit of the experimental data using Equation (4)). (**c**) Temperature-dependent K_eff_ values of the S1 and S2 nanoparticles.

It can be seen from Figure 10 that K_eff_ decreased almost linearly with increasing temperature. However, the K_eff_ values in S2 were always larger than those in S1 at the same temperature. This could be attributed mainly to the larger SG regions in S2 as discussed earlier. Hence, we suggest that the existence of SG regions enhanced the exchange anisotropy of the otherwise clean ferrimagnetic-ferrimagnetic core-shell interface. The larger the SG regions, the larger the interface exchange anisotropy and thus the larger the effective magnetic anisotropy. It is interesting to notice that under the same experimental conditions, M_s_ in S2 had smaller values than those in S1 (Figure 6) while K_eff_ in S2 had larger values than those in S1 (Figure 10). It is true that the larger SG regions in S2 could result in larger K_eff_ values, but the net spins of the SG regions were randomly oriented with different pinning strengths. Hence, it will be more difficult to align all or most of the SG regions along the applied field compared with the SG regions in S1. This will result in less net magnetization in S2 compared with S1.

### 3.4. Magnetic Hyperthermia Studies of Core-Shell Nanoparticles in Agar Gel Phantom

The heat conversion ability of the core-shell nanoparticles synthesized from the organometallic decomposition method was determined in agar gel phantom. Agar gel is the commonly used phantom for the magnetic hyperthermia and MRI studies of the nanoparticles for imaging and heating efficiency determination [60]. The agar gel is a heterogenous polysaccharide that consists of agarose and agaropectin polymers that adsorb huge amounts of water and form a three-dimensional network, which has tissue-mimicking properties. The ferrogel of the core-shell nanoparticles was prepared by adding 50 mg of the agar powder to the 1 mL of chitosan coated core-shell nanoparticles with a concentration of 5 mg/mL. This nano dispersion was sonicated for 10 min and heated to 95 °C in a water bath for 30 min. Upon heating, it formed a homogenous viscus liquid that was cooled to room temperature to obtain the agar ferrogel.

**Figure 11:**
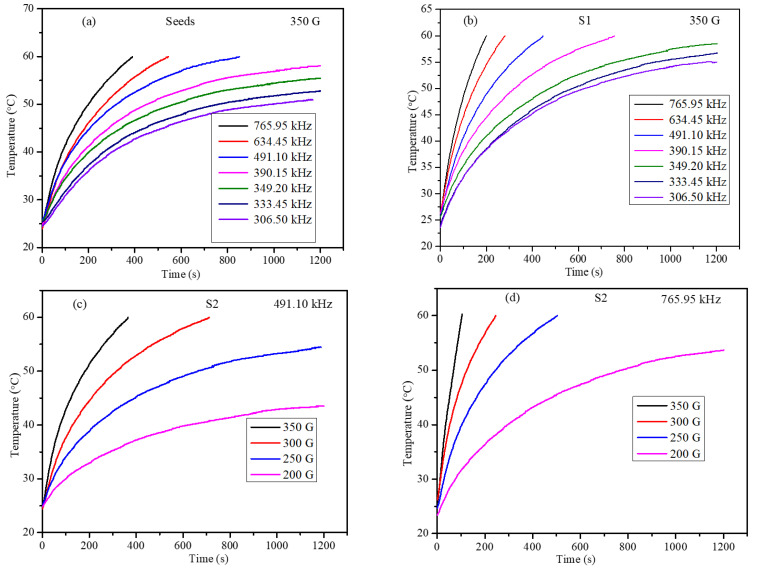
Heating profiles of ferrogel (**a**) CoFe_2_O_4_ seed nanoparticles at 350 G, (**b**) S1 core-shell ferrogel at 350 G, (**c**) S2 ferrogel under 491.10 kHz with field amplitude 200–350 G, and (**d**) S2 ferrogel 765.95 kHz, under 200–350 G magnetic field.

The heating efficiency values of the ferrogel phantoms were obtained using a nanoscale bio-magnet instrument at frequencies of 765.95, 634.45, 491.10, 390.15, 349.20, 333.45, and 306.50 kHz and field strengths of 200, 250, 300, and 350 G. The instrument parameters were well within the permissible levels of C = H × f = 5 × 10^9^ Am^−1^s^−1^ for use in human trials [60]. Heating profiles were recorded for the ferrogel until the temperature of the ferrogel reached 60 °C. The readings were taken for a maximum of 20 min exposure time when the ferrogel temperature did not exceed 60 °C. The heating profiles of the seeds and core-shell nanoparticle ferrogels are shown in Figure 11. The heating curves clearly demonstrated that the frequency and field strength of the alternating magnetic field (AMF) had significant effects on the magneto thermic efficiency of the core-shell nanoparticles. The heating profiles were obtained under identical conditions, and SAR values were determined from the initial slope of the heating curve using Equation (2). The critical requirement for the nanoparticle to be used for magnetic hyperthermia for killing cancer cells is that the particles should be able to increase the temperature above 42 °C at which cancer cells start showing a denaturation property. Under a low magnetic field and frequency, the nanoparticles were unable to increase the temperature above 42 °C, and these conditions were not considered for obtaining the SAR values.

**Figure 12:**
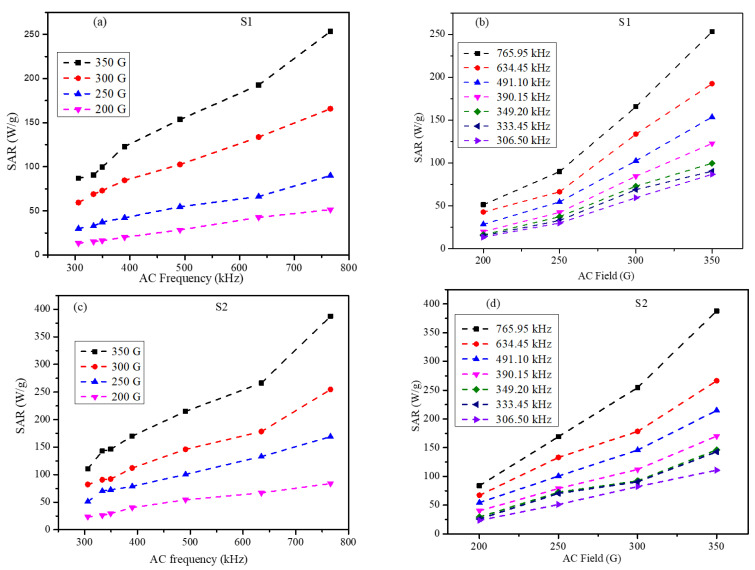
SAR values of the core-shell agar ferrogels (**a**) S1-SAR vs. field frequency, (**b**) S1-SAR vs. field amplitude, (**c**) S2-SAR vs. field frequency, and (**d**) S2-SAR vs. field amplitude.

The SAR values of the core-shell nanoparticle ferrogel phantoms obtained for the S1 and S2 nanoparticles are shown in Figure 12. The SAR values of S2 were larger than those for S1 at all frequency and field-strength parameters. The SAR values of the S1 nanoparticles increased linearly with the increase in frequency under 200–350 G field amplitude whereas the S2 particles showed similar behavior under lower field amplitude and frequency but showed a significant deviation under higher frequency and field strength. To understand the correlation between the applied field and heating rates, field-dependent SAR values were plotted and are shown in Figure 12b S1 and Figure 12d S2. For the S1 nanoparticles, the SAR values varied from 86.85–253.25 W/g in the frequency range of 306.5–765.85 kHz under 350 G AC field amplitude, while the SAR values dropped to 13.7–51.4 W/g in the same frequency range under the 200 G field amplitude; corresponding plots are shown in Figure 12a,b. The SAR values of the S2 nanoparticles are shown in Figure 12c,d; the SAR of the S2 nanoparticles also showed strong dependency on both the frequency and field strength of the AMF. The S2 nanoparticles had the highest SAR value of 387.6 W/g under 765.95 kHz and 350 G field amplitude but dropped to 23.4 W/g under 200 G and 306.5 kHz frequency. The high SAR value of the S2 nanoparticles is interesting because of the lower saturation magnetization of the S2 nanoparticles compared to the S1 particles.

**Figure 13:**
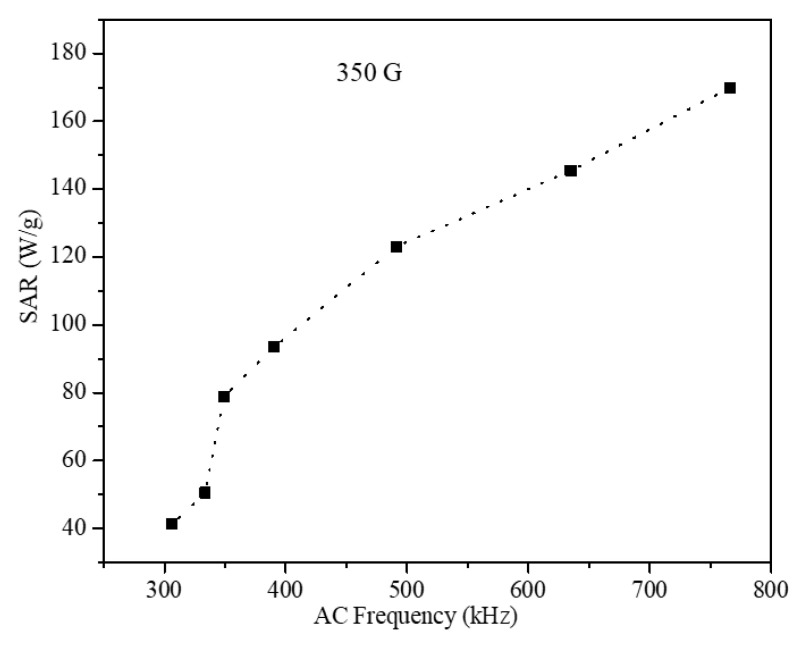
SAR values of CoFe_2_O_4_ seed nanoparticles obtained under 350 G field and 306.5–765.97 kHz frequencies.

To understand the effect of core-shell geometry on the SAR values, the magneto thermic effect of the CoFe_2_O_4_ seed nanoparticle ferrogel was obtained under similar conditions. The SAR values of the seed nanoparticles are shown in Figure 13. The SAR values of the seeds showed a nonlinear dependence on AMF frequency in the range of 306.5–368 kHz, whereas it increased linearly at higher frequency. The SAR values of the seed ferrogel were 170 and 40.4 W/g with field frequencies 765.95 and 306.5 kHz, respectively, under an AMF amplitude of 350 G. These values are low compared with the SAR values of S1 and S2 obtained under similar conditions. Table 3 displays the different parameters of the three samples.

From Figure 12, we can see that SAR values for both the S1 and S2 samples displayed a roughly linear dependency on the frequency. The field dependence of SAR showed a roughly linear behavior in S2 and a nonlinear behavior in S1. Thus, in both samples, the LRT was roughly applied in S1 but not in S2. The highest SAR values were 253.2 W/g and 387.6 W/g for S1 and S2, respectively. Across all AMF parameters (strength and frequency), the SAR values for S2 were larger than those for S1 under the same AMF parameters. We believe that this could be attributed to the larger effective anisotropy of S2 at all temperatures. Hence, it is very interesting to notice that hard–soft core-shell NPs can be tailored by introducing a controlled amount of SG regions to enhance the heating efficiency for magnetic hyperthermia applications.

## 4. Conclusions

The magnetic properties of hard–soft core-shell CoFe_2_O_4_-Fe_3_O_4_ nanoparticles were examined in the temperature range 5–300 K. Two sets of nanoparticles with average sizes of 9.5 ± 1.1 (S1) and 12.1 ± 1.7 (S2) nm were synthesized by the organometallic decomposition method. The core size (6 nm) was fixed in both samples with shell thicknesses of 3.5 and 6.1 nm for S1 and S2, respectively. The exchange bias displayed significant nonmonotonic temperature and field dependences in both samples, but they were more pronounced in S2 with the larger shell thickness. Notably, both samples displayed the exchange bias effect in the zero-field cooling state, but this was more pronounced in S2. The effective anisotropy constant was found to decrease almost linearly with temperature in both samples, but the magnitude in S2 was greater at all temperatures. The SAR values for both core-shell NPs displayed much larger values than those for the CoFe_2_O_4_ seed NPs, indicating the significant enhancement due to the core-shell structure. However, the SAR values for S2 were larger than those for S1 under the same field and frequency parameters. The larger effective anisotropy and SAR with smaller saturation magnetization in S2 are suggested to be due to the existence of interfacial spin-glass regions. The ability to tune magnetic properties and SAR values by controlling the core-shell structure of hard and soft magnetic phases makes this system a promising candidate for advanced hyperthermia applications.

## Figures and Tables

**Table 1:** Comparison of the lattice parameter with the reported values of the CoFe_2_O_4_ and Fe_3_O_4_ nanoparticles.

Nanoparticles	Lattice Parameter (Å)	
CoFe_2_O_4_	8.387	Ref [34]
Fe_3_O_4_	8.40	Ref [35]
CoFe_2_O_4_-seeds	8.376	This work
S1-Core-shell	8.420	This work
S2-Core-shell	8.390	This work

**Table 2:** (311) peak positions obtained for the individual CoFe_2_O_4_ and Fe_3_O_4_ nanoparticles and core-shell nanoparticles.

Particles	(311) Peak Position
Fe_3_O_4_	35.51
CoFe_2_O_4_	35.45
Mixture	35.48
S1-Core shell	35.30

**Table 3:** List of average sizes, effective anisotropy, and SAR values of the CoFe_2_O_4_ seed and core-shell nanoparticles.

Compositions	Average Sizes from XRD (nm)	Average TEM Sizes (nm)	Shell Thickness (nm)	M_S_ (emu/g)	K_eff_ 10^4^ J/m^3^	SAR (W/g) at 765.97 kHz and 350 G
CoFe_2_O_4_ seeds	6.9	6.0 ± 1.0		39.07	5.55	170.2
S1 [Fe_3_O_4_(CoFe_2_O_4_)	10.5	9.5 ± 1.1	3.5	69.97	1.23	253.2
S2 [Fe_3_O_4_(CoFe_2_O_4_)	13.5	12.1 ± 1.7	6.1	66.16	2.16	387.6

## Data Availability

Not applicable.
